# Author Correction: Sensory innervation in porous endplates by Netrin-1 from osteoclasts mediates PGE2-induced spinal hypersensitivity in mice

**DOI:** 10.1038/s41467-019-13970-0

**Published:** 2020-01-06

**Authors:** Shuangfei Ni, Zemin Ling, Xiao Wang, Yong Cao, Tianding Wu, Ruoxian Deng, Janet L. Crane, Richard Skolasky, Shadpour Demehri, Gehua Zhen, Amit Jain, Panfeng Wu, Dayu Pan, Bo Hu, Xiao Lyu, Yusheng Li, Hao Chen, Huabin Qi, Yun Guan, Xinzhong Dong, Mei Wan, Xuenong Zou, Hongbin Lu, Jianzhong Hu, Xu Cao

**Affiliations:** 1 Department of Orthopaedic Surgery, The Johns Hopkins University School of Medicine, Baltimore, MD 21205 USA; 2 Department of Spine Surgery, Xiangya Hospital, Central South University, Changsha, 410008 P. R. China; 3 Guangdong Provincial Key Laboratory of Orthopedics and Traumatology, Department of Spinal Surgery, The First Affiliated Hospital of Sun Yat-sen University, Guangzhou, 51008 P. R. China; 4 Department of Pediatrics, The Johns Hopkins University School of Medicine, Baltimore, MD 21205 USA; 5 Department of Radiology and Radiological Science, The Johns Hopkins University School of Medicine, Baltimore, MD 21205 USA; 6 Department of Anesthesiology and Critical Care Medicine, The Johns Hopkins University School of Medicine, Baltimore, MD 21205 USA; 7 Department of Neuroscience, Neurosurgery, and Dermatology, Center of Sensory Biology, The Johns Hopkins University School of Medicine, Howard Hughes Medical Institute, Baltimore, MD 21205 USA; 8 Department of Sports Medicine, Xiangya Hospital, Central South University, Changsha, 410008 P. R. China

**Keywords:** Chronic pain, Bone

Correction to: *Nature Communications* 10.1038/s41467-019-13476-9, published online 10 December 2019.

The original version of this Article contained an error in the spelling of the author Shadpour Demehri, which was incorrectly given as Shadpour Demehril, and the author Richard Skolasky which was incorrectly given as Richard Skolask. This has now been corrected in both the PDF and HTML versions of the Article.

