# Supplementary material for: Sensory innervation in porous endplates by Netrin-1 from osteoclasts mediates PGE2-induced spinal hypersensitivity in mice
Source: Nat Commun. 2019 Dec 10;10:5643. doi: 10.1038/s41467-019-13476-9 (PMC6904550; doi:10.1038/s41467-019-13476-9)
Supplement: Supplementary file 1 — Supplementary Information [file 41467_2019_13476_MOESM1_ESM.pdf]

Sensory innervation in porous endplates by netrin-1 from osteoclasts mediates PGE2-induced spinal hypersensitivity in mice

Ni et al.

Supplementary Figures and legends

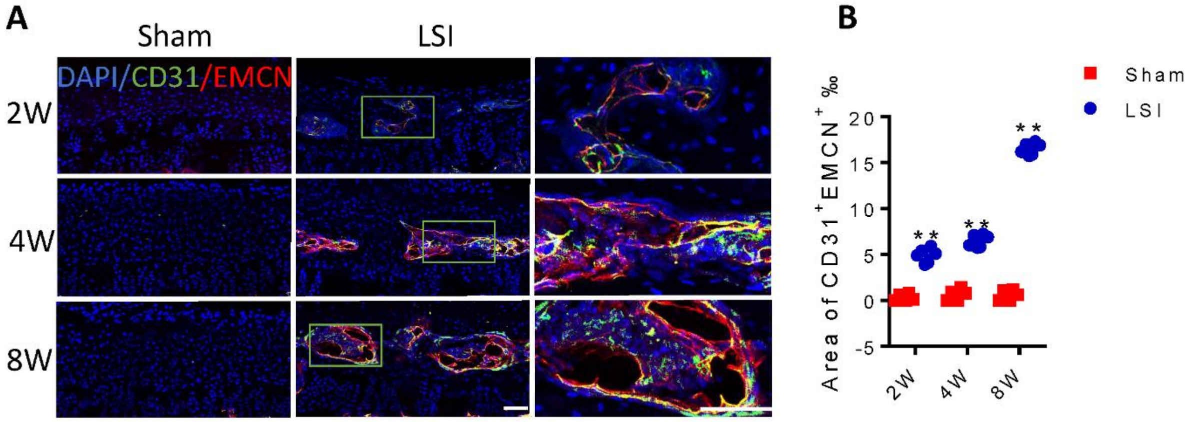

Supplementary fig. 1 The formation of CD31^{+}Emcn^{+} vessels in Endplate during spinal instability. (a) Representative images of immunofluorescent analysis of CD31^{+} (green), Emcn^{+} (red) and DAPI (blue) staining of nuclei in mouse caudal endplates of L4/5 at 2, 4 and 8 weeks after LSI or sham surgery. Scale bars, 50 μm. (b) Percentage of CD31^{+} Emcn^{+} area in endplates. **$p < 0.01$ compared with the sham surgery mice at the corresponding time points. n = 6 per group. Statistical significance was determined by multifactorial ANOVA, and all data are shown as means ± standard deviations. Source data are provided as a Source Data file.

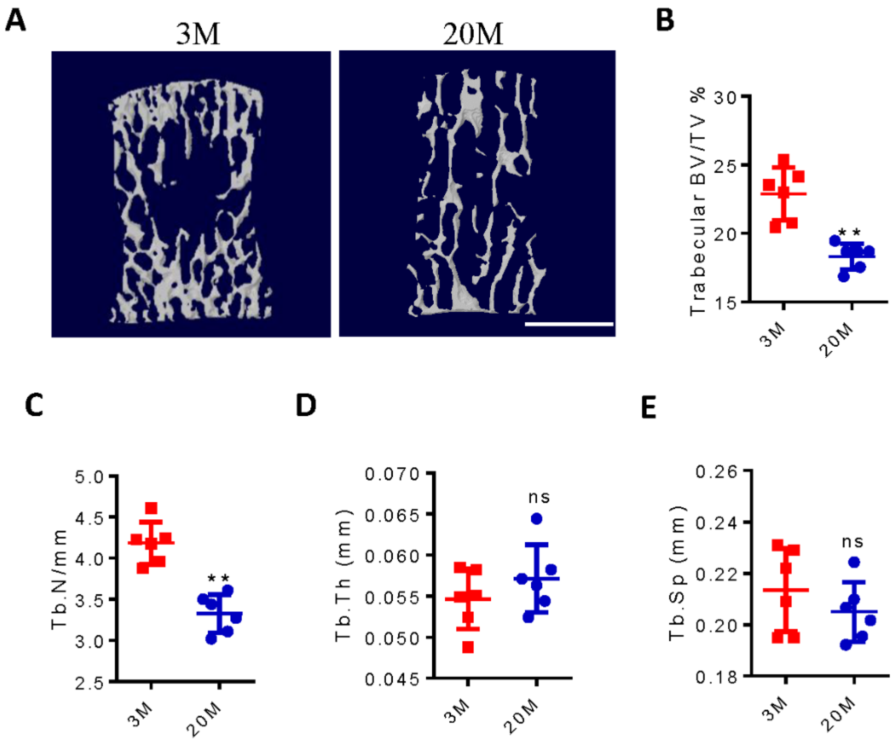

Supplementary fig. 2 The µCT analysis of the vertebral trabecular bone during aging. (a) Representative three-dimensional high-resolution µCT images of the trabecular bone of L5 vertebrae (coronal view) in 3-month-old and 20-month-old mice. Scale bars, 1 mm. (b-e) Quantitative analysis of the trabecular bone volume/total volume (BV/TV) (b) and trabecular bone number (Tb.N, c), trabecular bone thickness (Tb.Th, d), and trabecular bone separation distribution (Tb. Sp, e) in L5 vertebrae determined by µCT. **$p < 0.01$, ns, no significant difference compared with the 3-month-old mice. n = 6 per group (b-e). Statistical significance was determined by two-tailed Student's t-test, and all data are shown as means ± standard deviations. Source data are provided as a Source Data file.

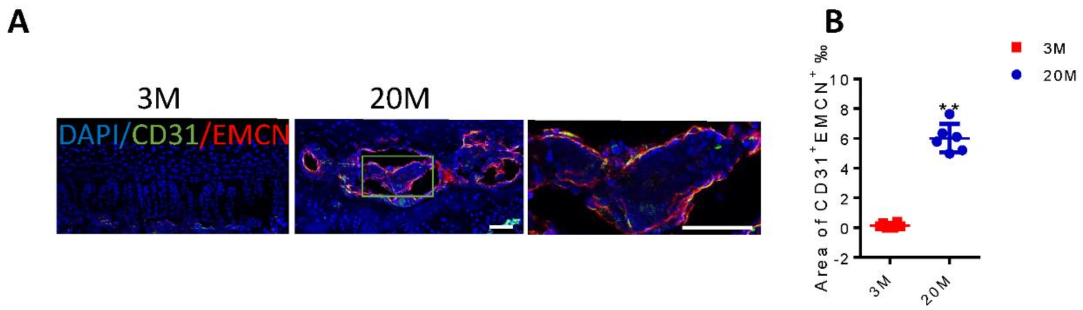

Supplementary fig. 3 The formation of CD31$^+$Emcn$^+$ vessels in Endplate during aging. (a) Representative images of immunofluorescent analysis of CD31$^+$ (green), Emcn$^+$ (red) and DAPI (blue) staining of nuclei in mouse caudal endplates of L4/5 in 20-month-old and 3-month-old mice. Scale bars, 50 µm. (b) Quantitative analysis of the percentage of CD31$^+$Emcn$^+$ area in endplates. **$p < 0.01$ compared with the 3-month-old mice. n = 6 per group. Statistical significance was determined by two-tailed Student's t-test, and all data are shown as means ± standard deviations. Source data are provided as a Source Data file.

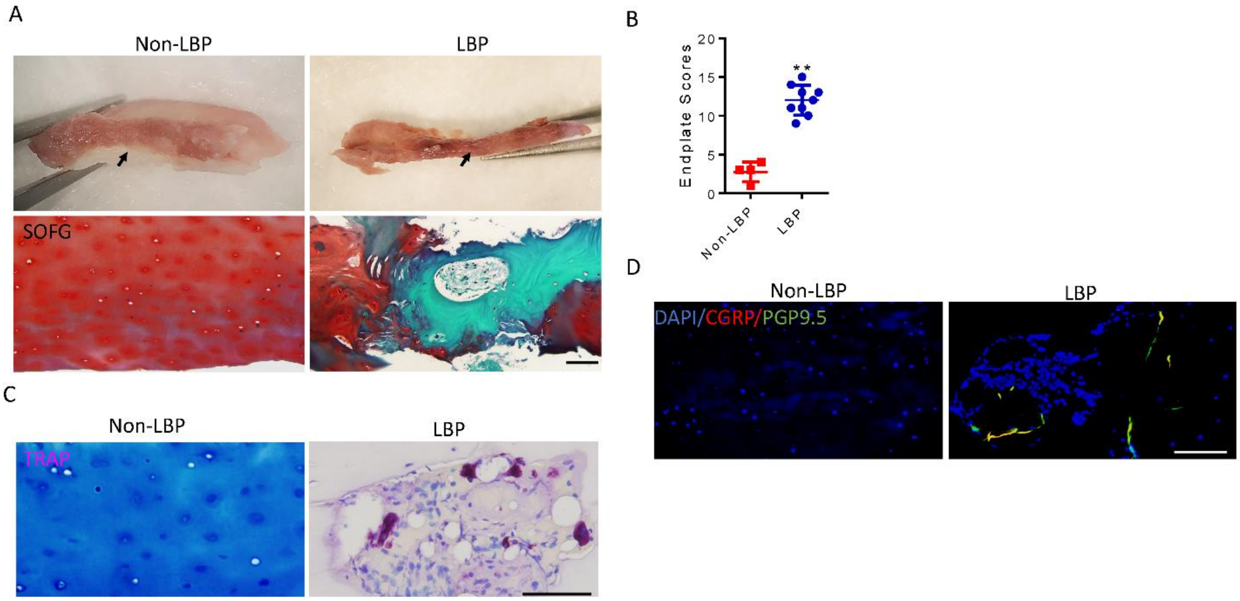

Supplementary fig. 4 Nerve innervation in the human sclerotic endplates. (a) Representative gross appearance (top) and images of safranin O and fast green staining of coronal sections (bottom) of the endplates from patients without LBP or with LBP. Scale bars, 50 μm. (b) Endplate scores of the samples from patients without LBP or with LBP. (c) Representative images of TRAP (magenta) staining of coronal sections of the endplates from patients without LBP or with LBP. Scale bars, 50 μm. (d) Representative immunofluorescent images of $CGRP^+$ (red), $PGP9.5^+$ (green) and DAPI (blue) staining of nuclei in the endplates. Scale bars, 50 μm. $^{**}p < 0.01$ compared with patients without LBP. n = 4 of non-LBP group, n=9 of LBP group. LBP: low back pain. Statistical significance was determined by two-tailed Student's t-test, and all data are shown as means ± standard deviations. Source data are provided as a Source Data file.

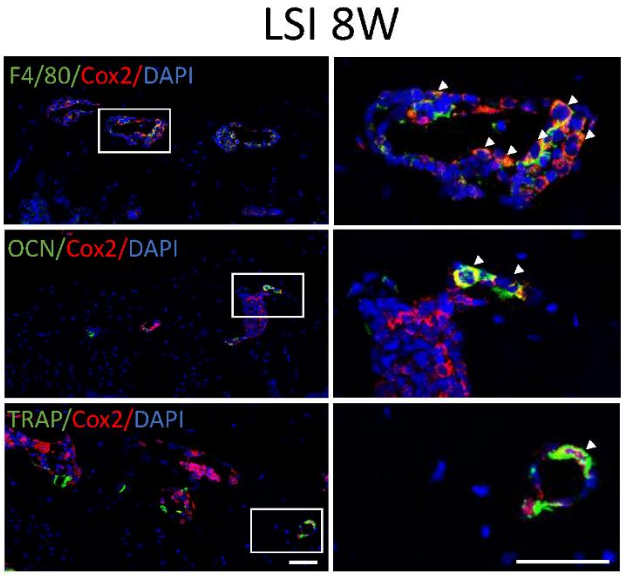

Supplementary fig. 5 The potential source of PGE2 in porous endplates. Representative immunofluorescent images of $cox2^+$ (red) and $F4/80^+$ (green), $cox2^+$ (red) and OCN^+ (green), $cox2^+$ (red) and $TRAP^+$ (green) and DAPI (blue) staining of nuclei in the endplates. Scale bars, 50 μm.

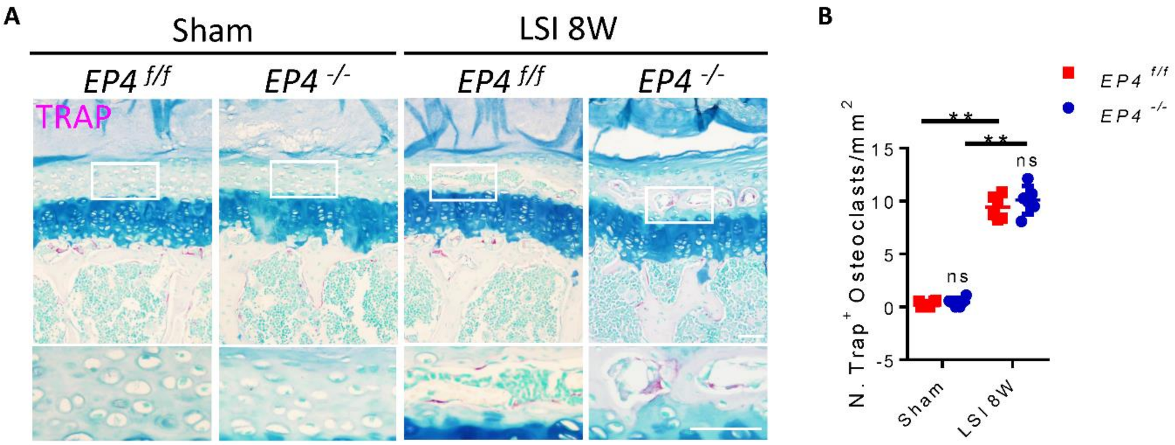

Supplementary fig. 6 EP4 knockout did not affect the LSI-induced increase in the number of TRAP⁺ osteoclasts in endplates. (a) Representative images of coronal mouse caudal endplate sections of L4/5 stained for TRAP (magenta) at 8 weeks after LSI or sham surgery in $EP4^{f/f}$ and $EP4^{-/-}$ mice. Scale bars, 50 μm. (b) Quantitative analysis of the number of $TRAP^+$ cells in endplates. **p < 0.01 compared with sham surgery group. ns, no significant difference compared with sham surgery mice in the corresponding transgenic group. n = 6 per group. Statistical significance was determined by multifactorial ANOVA, and all data are shown as means ± standard deviations. Source data are provided as a Source Data file.

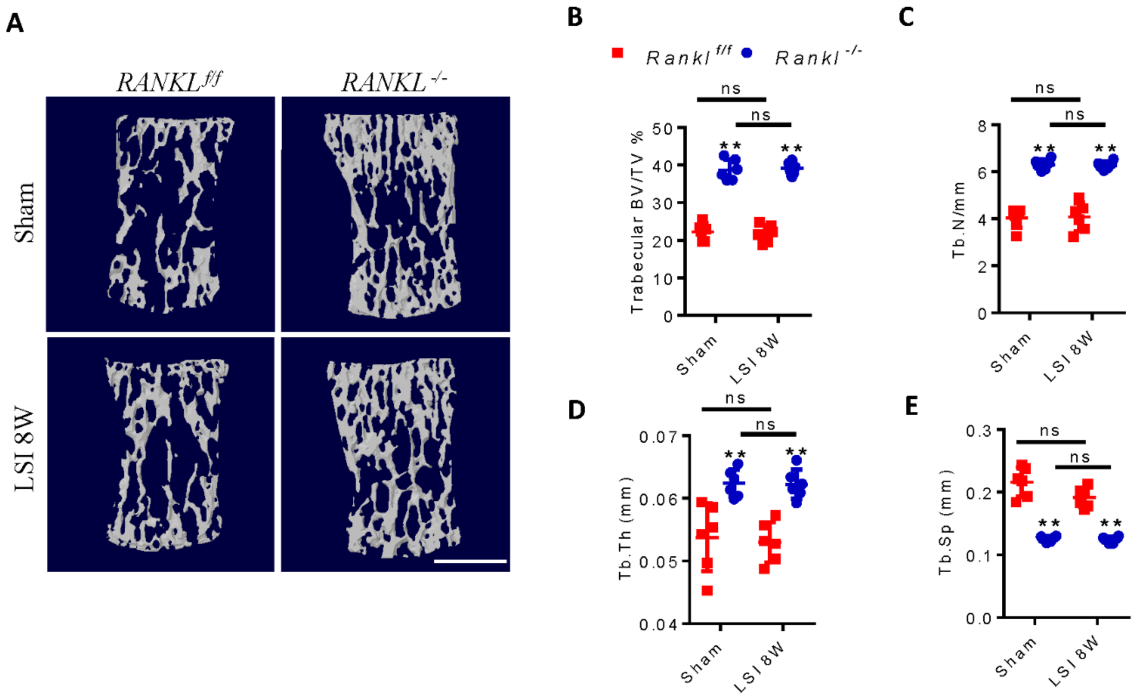

Supplementary fig. 7 The osteopetrotic phenotype of Vertebrae in *Rankl*$^{-/-}$ mice. (a) Representative µCT images of the trabecular bone (coronal view) in L5 vertebrae of *Rankl*$^{-/-}$ and *Rankl*$^{f/f}$ mice in sham or LSI surgery group. Scale bars, 1 mm. (b-e) Quantitative analysis of the Trabecular BV/TV (b), Tb.N (c), Tb.Th (d), and Tb. Sp (e) of the mouse L5 vertebrae determined by µCT. BV, Bone Volume. TV, Total Volume. Tb.N, Trabecular bone Number. Tb.Th, Trabecular bone Thickness. Tb. Sp, Trabecular Separation distribution. **p < 0.01 compared with *Rankl*$^{f/f}$ mice. ns, no significant difference compared with sham surgery mice in the corresponding transgenic group. n = 6 per group (b-e). Statistical significance was determined by multifactorial ANOVA, and all data are shown as means ± standard deviations. Source data are provided as a Source Data file.

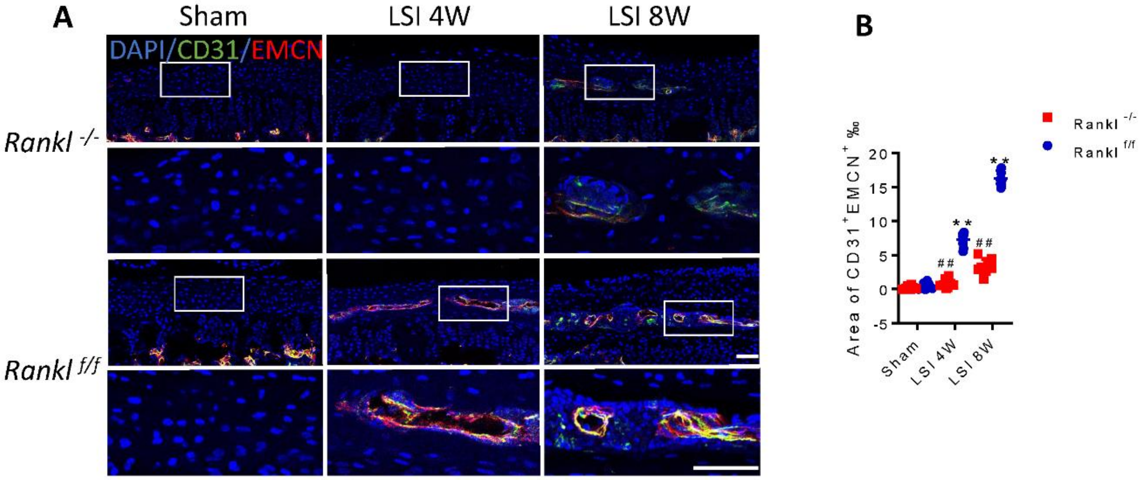

Supplementary fig. 8 Decreased osteoclasts activity diminished the formation of CD31^{+}Emcn^{+} vessels in Endplate. (a) Representative images of immunofluorescent analysis of CD31^{+} (green), Emcn^{+} (red) and DAPI (blue) staining of nuclei in caudal endplates of L4/5 in *Rankl*$^{-/-}$ or *Rankl*$^{f/f}$ mice at 4 and 8 weeks after LSI or sham surgery. Scale bars, 50 µm. b) Quantitative analysis of the percentage of CD31^{+}Emcn^{+} area in caudal endplates. $^{**}p < 0.01$ compared with *Rankl*$^{f/f}$ sham surgery mice, $^{\#\#}p < 0.01$ compared with *Rankl*$^{f/f}$ LSI surgery mice at the corresponding time points. n = 8 per group. Statistical significance was determined by multifactorial ANOVA, and all data are shown as means ± standard deviations. Source data are provided as a Source Data file.

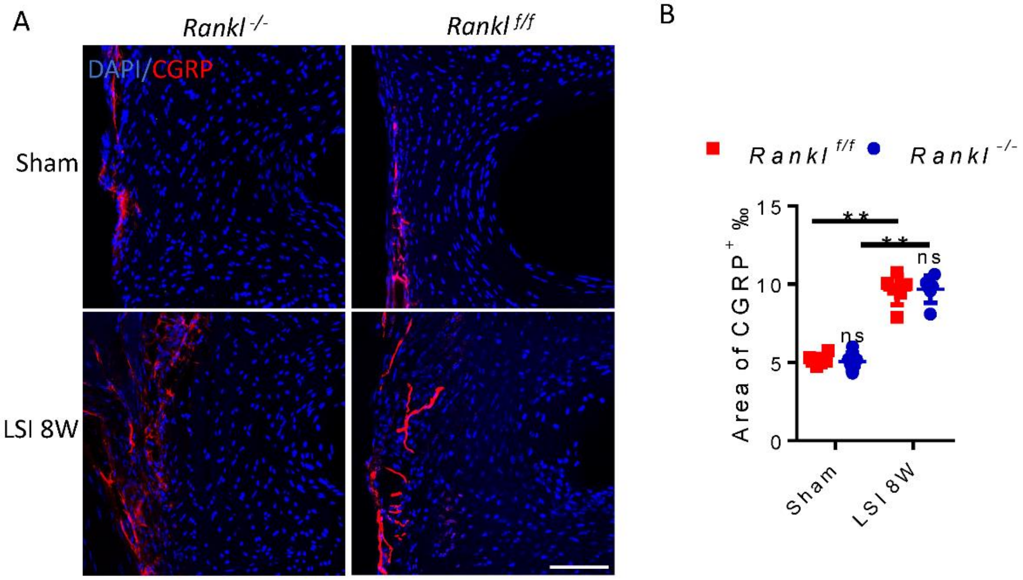

Supplementary fig. 9 Reduction of osteoclast activity did not inhibit sensory innervation in the annulus fibrosus in LSI mice. (a) Representative immunofluorescent images of CGRP^{+} sensory nerve fibers (red) and DAPI (blue) staining of nuclei in mouse annulus fibrosus of L4/5 at 8 weeks after LSI or sham surgery. Scale bars, 100 µm. (b) Percentage of CGRP^{+} area in annulus fibrosus. **p < 0.01 compared with sham surgery mice, ns, no significant difference, compared with *Rankl*$^{f/f}$ LSI surgery mice. n = 6 per group. Statistical significance was determined by multifactorial ANOVA, and all data are shown as means ± standard deviations. Source data are provided as a Source Data file.

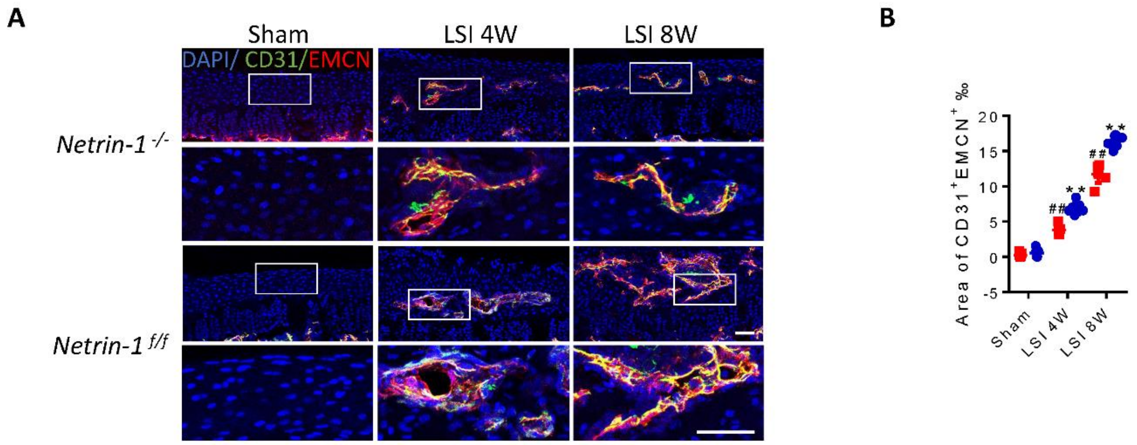

Supplementary fig. 10 Knockout of netrin-1 in the TRAP+ lineage cells inhibited the formation of CD31$^+$Emcn$^+$ vessels in Endplate. (a) Representative images of immunofluorescent analysis of CD31$^+$ (green), Emcn$^+$ (red) and DAPI (blue) staining of nuclei in caudal endplates of L4/5 in *Netrin-1*$^{-/-}$ or *Netrin-1*$^{f/f}$ mice at 4 and 8 weeks after LSI or sham surgery. Scale bars, 50 μm. (b) Quantitative analysis of the percentage of CD31$^+$Emcn$^+$ area in caudal endplate. $^{**}p < 0.01$ compared with *Netrin-1*$^{f/f}$ sham surgery mice, $^{\#\#}p < 0.01$ compared with *Netrin-1*$^{f/f}$ LSI surgery mice at the corresponding time points. n = 7 per group. Statistical significance was determined by multifactorial ANOVA, and all data are shown as means ± standard deviations. Source data are provided as a Source Data file.

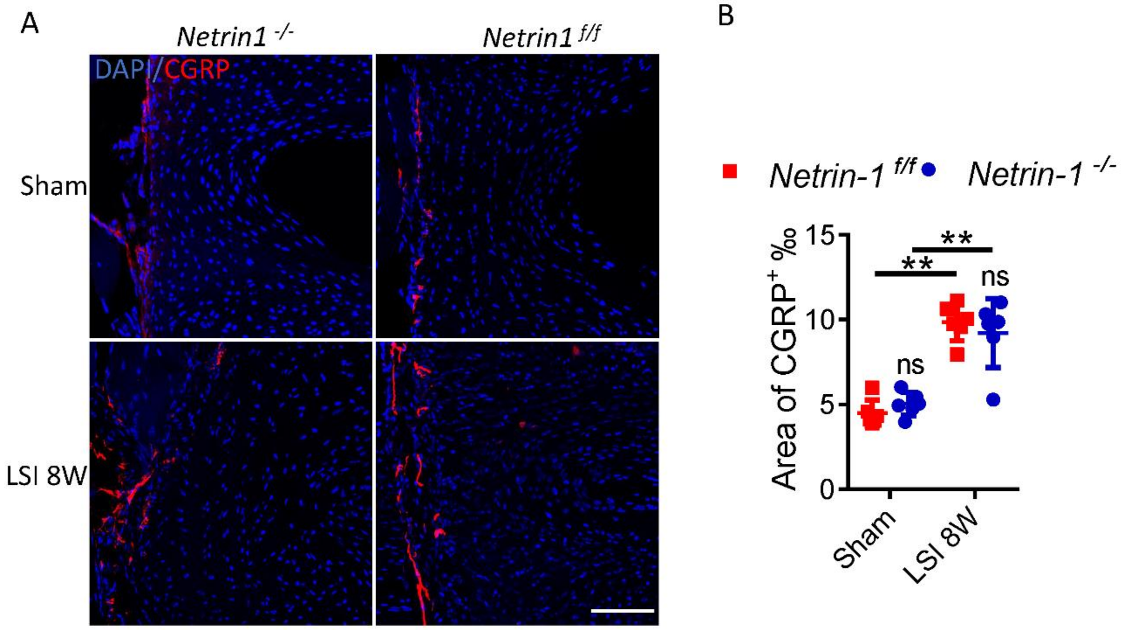

Supplementary fig. 11 Knockout of Netrin-1 in the Trap$^+$ cells did not inhibit sensory innervation in the annulus fibrosus in LSI mice. (a) Representative immunofluorescent images of CGRP$^+$ sensory nerve fibers (red) and DAPI (blue) staining of nuclei in mouse annulus fibrosus of L4/5 at 8 weeks after LSI or sham surgery. Scale bars, 100 μm. (b) Percentage of CGRP$^+$ area in annulus fibrosus. **p < 0.01 compared with sham surgery mice, ns, no significant difference, compared with *Netrin-1$^{f/f}$* LSI surgery mice. n = 6 per group. Statistical significance was determined by multifactorial ANOVA, and all data are shown as means ± standard deviations. Source data are provided as a Source Data file.

Supplementary Tables

Supplementary Table 1. Information for the human samples

	No-Low Back Pain	Frequent Low Back Pain
Sample Size	4	9
Age (Years)	28.3±3.5	56.6±5.4
Sex (Male/Female)	3/1	5/4
Body Mass Index (BMI)	22.5±1.9	23.9±2.4
Disc Level	L4/5(3)/L5/S1(1)	L3/4(2)/L4/5(4)/L5/S1(3)
Endplate lesions	N/A	8
Pfirrmann grading (grade 3/grade 4)	1/3	4/5

Supplementary Table 2. The primers sequence for qRT-PCR

Target gene	Forward primer (5'-3')	Reverse primer (5'-3')
PGE synthase (PGES)	TTTCTGCTCTGCAGCACACT	GATTGTCTCCATGTCGTTGC
cox-2	CAGACAACATAAACTGCGCCTT	GATACACCTCTCCACCAATGACC
IL-1β	TTCAGGCAGGCAGTATCACTC	CGTCACACACCAGCAGGTTAT
IL-17	TCTCCACCGCAATGAAGACC	CACACCCACCAGCATCTTCT
IL-2	TTGTGCTCCTTGTCAACAGC	CTGGGGAGTTTCAGGTTCCT
TNF-α	ATGAGCACAGAAAGCATGA	AGTAGACAGAAGAGCGTGGT
GAPDH	AATGTGTCCGTCGTGGATCTGA	AGTGTAGCCCAAGATGCCCTTC
