# Supplementary material for: Sensory innervation in porous endplates by Netrin-1 from osteoclasts mediates PGE2-induced spinal hypersensitivity in mice
Source: Nat Commun. 2019 Dec 10;10:5643. doi: 10.1038/s41467-019-13476-9 (PMC6904550; doi:10.1038/s41467-019-13476-9)
Supplement: Supplementary file 3 — Reporting Summary [file 41467_2019_13476_MOESM3_ESM.pdf]

nature research

Corresponding author(s): Xu Cao

Last updated by author(s): Jun 10, 2019

Reporting Summary

Nature Research wishes to improve the reproducibility of the work that we publish. This form provides structure for consistency and transparency in reporting. For further information on Nature Research policies, see Authors & Referees and the Editorial Policy Checklist.

Statistics

For all statistical analyses, confirm that the following items are present in the figure legend, table legend, main text, or Methods section.

n/a	Confirmed	
☐	☒	The exact sample size (n) for each experimental group/condition, given as a discrete number and unit of measurement
☐	☒	A statement on whether measurements were taken from distinct samples or whether the same sample was measured repeatedly
☐	☒	The statistical test(s) used AND whether they are one- or two-sided<br>*Only common tests should be described solely by name; describe more complex techniques in the Methods section.*
☒	☐	A description of all covariates tested
☐	☒	A description of any assumptions or corrections, such as tests of normality and adjustment for multiple comparisons
☐	☒	A full description of the statistical parameters including central tendency (e.g. means) or other basic estimates (e.g. regression coefficient) AND variation (e.g. standard deviation) or associated estimates of uncertainty (e.g. confidence intervals)
☐	☒	For null hypothesis testing, the test statistic (e.g. F, t, r) with confidence intervals, effect sizes, degrees of freedom and P value noted<br>*Give P values as exact values whenever suitable.*
☒	☐	For Bayesian analysis, information on the choice of priors and Markov chain Monte Carlo settings
☒	☐	For hierarchical and complex designs, identification of the appropriate level for tests and full reporting of outcomes
☒	☐	Estimates of effect sizes (e.g. Cohen's d, Pearson's r), indicating how they were calculated

Our web collection on statistics for biologists contains articles on many of the points above.

Software and code

Policy information about availability of computer code

Data collection	µCT SCAN: Skyscan 1172. ImageJ (NIH) software, SPSS
Data analysis	µCT images were reconstructed and analyzed using NRecon v1.6 and CTAn v1.9 (Skyscan US, San Jose, CA). We analyzed endplate-related parameters using 3-dimensional model visualization software, CTVol v2.0 (Skyscan US). All data analyses were performed using SPSS, version 15.0, software (IBM Corp.). ImageJ (NIH) software was used for quantitative analysis of histology.

For manuscripts utilizing custom algorithms or software that are central to the research but not yet described in published literature, software must be made available to editors/reviewers. We strongly encourage code deposition in a community repository (e.g. GitHub). See the Nature Research guidelines for submitting code & software for further information.

Data

Policy information about availability of data

All manuscripts must include a data availability statement. This statement should provide the following information, where applicable:

- Accession codes, unique identifiers, or web links for publicly available datasets
- A list of figures that have associated raw data
- A description of any restrictions on data availability

"Statistics" of "Materials and Methods"

Field-specific reporting

Please select the one below that is the best fit for your research. If you are not sure, read the appropriate sections before making your selection.

☒ Life sciences ☐ Behavioural & social sciences ☐ Ecological, evolutionary & environmental sciences

For a reference copy of the document with all sections, see nature.com/documents/nr-reporting-summary-flat.pdf

Life sciences study design

All studies must disclose on these points even when the disclosure is negative.

Sample size	Figure legends and supplementary materials
Data exclusions	All inclusion/ exclusion criteria were pre-established and no samples or animals were excluded from the analysis
Replication	"Statistics" of "Materials and Methods",
Randomization	The experiments were randomized.
Blinding	The experiments were randomized, and the investigators were blinded to allocation during experiments and outcome assessment. All behavioral tests were performed by the same investigator, who was blinded to the study groups.

Reporting for specific materials, systems and methods

We require information from authors about some types of materials, experimental systems and methods used in many studies. Here, indicate whether each material, system or method listed is relevant to your study. If you are not sure if a list item applies to your research, read the appropriate section before selecting a response.

Materials & experimental systems

n/a	Involved in the study
☐	☒ Antibodies
☒	☐ Eukaryotic cell lines
☒	☐ Palaeontology
☐	☒ Animals and other organisms
☒	☐ Human research participants
☒	☐ Clinical data

Methods

n/a	Involved in the study
☒	☐ ChIP-seq
☒	☐ Flow cytometry
☒	☐ MRI-based neuroimaging

Antibodies

Antibodies used	The supplier name and catalog number are Included in "Histochemistry", "immunohistochemistry", "histomorphometry", and "western blot" of "Methods"
Validation	validation statements on the manufacturer's website

Animals and other organisms

Policy information about studies involving animals; ARRIVE guidelines recommended for reporting animal research

Laboratory animals	The laboratory animals are reported in "Mice " of "Methods" and figure legends
Wild animals	We purchased C57BL/6J (WT) male mice from Charles River Laboratories (Wilmington, MA). The detailed information are included in the "Methods"
Field-collected samples	All the relevant parameters are included in "Mice " of "Methods" and figure legends
Ethics oversight	All mice were maintained at the animal facility of The Johns Hopkins University School of Medicine. All experimental protocols (M018M264) were approved by the Animal Care and Use Committee of The Johns Hopkins University, Baltimore, MD.

Note that full information on the approval of the study protocol must also be provided in the manuscript.
